# Assessment of liver cirrhosis severity with extracellular volume fraction MRI

**DOI:** 10.1038/s41598-022-13340-9

**Published:** 2022-06-08

**Authors:** Narine Mesropyan, Patrick A. Kupczyk, Leona Dold, Michael Praktiknjo, Johannes Chang, Alexander Isaak, Christoph Endler, Dmitrij Kravchenko, Leon M. Bischoff, Alois M. Sprinkart, Claus C. Pieper, Daniel Kuetting, Christian Jansen, Ulrike I. Attenberger, Julian A. Luetkens

**Affiliations:** 1 Department of Diagnostic and Interventional Radiology and Quantitative Imaging Lab Bonn (QILaB), University Hospital Bonn, Venusberg-Campus 1, 53127 Bonn, Germany; 2 Department of Internal Medicine I and Center for Cirrhosis and Portal Hypertension Bonn (CCB), University Hospital Bonn, Venusberg-Campus 1, 53127 Bonn, Germany

**Keywords:** Medical research, Biomarkers, Gastroenterology, Hepatology, Biomarkers, Diagnostic markers, Predictive markers, Prognostic markers

## Abstract

We aimed to investigate the diagnostic utility of MRI extracellular volume fraction (ECV) for the assessment of liver cirrhosis severity as defined by Child–Pugh class. In this retrospective study, 90 patients (68 cirrhotic patients and 22 controls), who underwent multiparametric liver MRI, were identified. Hepatic T1 relaxation times and ECV were assessed. Clinical scores of liver disease severity were calculated. One-way analysis of variance (ANOVA) followed by Tukey’s multiple comparison test, Spearman’s correlation coefficient, and receiver operating characteristic (ROC) analysis were used for statistical analysis. In cirrhotic patients, hepatic native T1 increased depending on Child–Pugh class (620.5 ± 78.9 ms (Child A) vs. 666.6 ± 73.4 ms (Child B) vs. 828.4 ± 91.2 ms (Child C), *P* < 0.001). ECV was higher in cirrhotic patients compared to the controls (40.1 ± 11.9% vs. 25.9 ± 4.5%, *P* < 0.001) and increased depending of Child–Pugh class (33.3 ± 6.0% (Child A) vs. 39.6 ± 4.9% (Child B) vs. 52.8 ± 1.2% (Child C), *P* < 0.001). ECV correlated with Child–Pugh score (r = 0.64, *P* < 0.001). ECV allowed differentiating between Child–Pugh classes A and B, and B and C with an AUC of 0.785 and 0.944 (*P* < 0.001, respectively). The diagnostic performance of ECV for differentiating between Child–Pugh classes A and B, and B and C was higher compared to hepatic native T1 (AUC: 0.651 and 0.910) and MELD score (AUC: 0.740 and 0.795) (*P* < 0.05, respectively). MRI-derived ECV correlated with Child–Pugh score and had a high diagnostic performance for the discrimination of different Child–Pugh classes. ECV might become a valuable non-invasive biomarker for the assessment of liver cirrhosis severity.

## Introduction

Although the burden and underlying causes of chronic liver disease (CLD) and cirrhosis vary worldwide, they are—with an increasing incidence—a major cause of morbidity and mortality^1–3^. Regardless of the pattern and underlying etiology, liver cirrhosis is characterized by severe scarring of the liver tissue with collagen deposition, architecture distortion and failed function, and is related to life-threatening complications such as portal hypertension, spontaneous bacterial peritonitis, ascites, variceal bleeding, hepatic encephalopathy, and hepatorenal syndrome. Outcome prediction of cirrhotic patients, who undergo surgery/interventions as well as overall mortality risk estimation are of great clinical importance. Therefore, different scores for the assessment of short- or long-term mortality, also for a specific etiology of chronic liver disease have been developed and proposed (e.g., MELD score or CLIF-C ACLF score). One of the most validated and widely used scoring systems, however, the Child–Pugh score, is simple to calculate and suitable for various etiologies of liver disease^4^. For instance, patients with a Child–Pugh A class have a generally good prognosis, and are considered for elective surgery. Patients with a Child–Pugh B class have an increased risk and commonly have to undergo medical optimization before surgery. For patients with a Child–Pugh C class elective surgery is contraindicated, as they have a mortality risk up to 82%^4–6^.

Imaging plays an important role for prognosis estimation and complication assessment in patients with CLD and cirrhosis. In this regard, magnetic resonance imaging (MRI) has experienced a steady evolution and is considered today the clinical standard in patients with CLD and cirrhosis, mainly for malignancy exclusion. Furthermore, current state-of-the art MRI techniques allow not only for morphological liver parenchyma assessment, but also for the assessment of liver function. Particularly, several MRI techniques such as diffusion-weighted imaging (DWI), as well as contrast-enhanced MRI have already been described in previous studies^7–11^. Another promising technique is quantitative T1 mapping with calculation of the extracellular volume fraction (ECV). The ability of T1 mapping with ECV calculation in liver fibrosis assessment has already been sufficiently described in patients with CLD of different etiologies as well as in animal models^12–16^. However, to our knowledge, the ability of MRI-derived ECV to assess liver cirrhosis severity has not been under investigation yet. The implementation of new non-invasive imaging-based biomarkers, which allows for comprehensive liver assessment beyond morphology (e.g., fibrosis quantification and possibly also liver function) and, at the same time, reproducible and simple to estimate, are highly desirable.

Therefore, the aim of this study was to investigate the diagnostic utility of MRI-derived ECV for the assessment of cirrhosis severity as well as to differentiate between different Child–Pugh classes in patients with CLD of various etiologies.

## Materials and methods

This retrospective study was approved by the local institutional review board that waived informed consent. Between January 2019 and September 2020 patients with confirmed diagnosis of liver cirrhosis, who underwent multiparametric liver MRI, were identified. The diagnosis of liver cirrhosis was established based on previous medical history, clinical examinations, liver biopsy as well as imaging according to the current guidelines^17^. Additionally, all patients with liver cirrhosis were categorized into three groups based on Child–Pugh classes of cirrhosis severity: A, B and C. Child–Pugh classes were calculated as a sum of individual points of clinical and laboratory criteria as previously published^18^. For patients with cholestatic liver disease, a modified Child–Pugh score was used. Patients with no history of chronic liver disease, who underwent clinical MRI examinations, were also enrolled into this study as a control group. The absence of chronic liver disease was based on previous medical history, clinical and laboratory tests. Exclusion criteria were contraindications to contrast-enhanced MRI and insufficient imaging quality. Laboratory markers were retrieved from the patients´ charts. Model of End-Stage Liver Disease (MELD) was also calculated.

### Magnetic resonance imaging

All MRI examinations were conducted on a clinical whole-body 1.5 Tesla MR-system (Ingenia, Philips Healthcare). A 32-channel body coil with digital interface was used for signal reception. Besides morphological sequences, hepatic T1 mapping before and 10 min after contrast media application was performed in the same slice position in end-expiration^19^. For T1 mapping, a heart rate independent 10-(2)-7-(2)-5-(2)-3-(2) modified Look-Locker inversion recovery (MOLLI) acquisition scheme with internal triggering was applied. Technical parameters were as follows: TR/TE 1.92/0.84 ms, FA 20°, parallel imaging factor 2, acquired voxel size 1.98 × 2.45 × 10.00 mm^3^, reconstructed voxel size 1.13 × 1.13 × 100.00 mm^3^, scan duration/breath hold 14 s. A gadolinium-based extracellular contrast agent in a dose of 1.0 mmol/ml solution with 0.1 mmol per kilogram of body weight (gadobutrol, Gadovist, Bayer Healthcare Pharmaceuticals) was administered as a single bolus with an injection rate of 1.5 ml/s.

### Image analysis

Image analysis was performed in consensus by two board-certified radiologists with 9 (J.A.L.) and 10 (P.K.) years of experience in abdominal radiology. The radiologists were blinded to the clinical data. The mean relaxation time of at least three representative regions of interest (ROI) drawn centrally in the right and left lobe at the level of portal vein bifurcation was used for the final analysis as previously described^13,14^. T1 values of the blood pool were obtained from the abdominal aorta from the same level. Calculation of ECV was performed with ROI-based values using following equation^20^: ECV = (1 − hematocrit) × (ΔR1_liver_/ΔR1_blood_), where R1 = 1/T1. Hematocrit was retrieved at the same day of MRI.

### Statistical analysis

Prism 8 (GraphPad Software) and SPSS Statistics (Version 25, IBM) were used for statistical analysis. Data were checked for normal distribution using the Shapiro–Wilk test. Data are given as mean ± standard deviation or absolute frequencies, as appropriate. Spearman’s correlation coefficient was used for a correlation analysis. One-way analysis of variance (ANOVA) followed by Tukey´s multiple comparison tests was performed to compare variables between groups of patients with liver cirrhosis of different Child–Pugh classes and control subjects. Dichotomous variables were compared by using the χ^2^ test. Receiver operating analysis (ROC) was used to determine the cut-offs with the highest combined sensitivity and specificity, positive predictive values (PPV), negative predictive values (NPV) and accuracy to differentiate between Child–Pugh classes A and B as well as Child–Pugh classes B and C. The level of statistical significance was set to *P* < 0.05.

### Ethical approval and informed consent

The presented study was approved by the institutional review board of the University of Bonn and hence all methods were performed in compliance with the ethical standards set in the 1964 Declaration of Helsinki as well as its later amendments. The requirement for written informed consent was waived by the institutional review board of the University of Bonn.

## Results

### Cohort characteristics

Sixty-eight patients (mean age: 55 ± 13 years; body mass index: 24.3 ± 3.8 kg/m^2^; 27 female) with liver cirrhosis were analyzed. N = 27 (39.7%), n = 32 (47.1%), and n = 9 (13.2%) of patients with liver cirrhosis had Child–Pugh class A, B and C, respectively. The etiologies of CLD and cirrhosis in the whole study cohort were as follows: alcoholic liver disease (n = 26, 38.2%); autoimmune liver diseases, including autoimmune hepatitis, primary sclerosing cholangitis, overlap syndromes, and primary biliary cirrhosis (n = 16, 23.5%); viral hepatitis (n = 8, 11.8%); non-alcoholic fatty liver disease (n = 3, 4.4%), and other unknown and/or rare etiologies (n = 15, 22.1%). Twenty-two patients (mean age: 46 ± 16 years ; body mass index: 25.6 ± 5.0 kg/m^2^; 8 female) without history of chronic liver disease, who had normal liver function tests were included as control subjects. The group of patients consisted of patients with clinical indications for liver MRI such as non-specific abdominal pain (9/22, 41%) and benign liver lesion characterization/follow-up (13/22, 59%). Clinical scores for the assessment of liver fibrosis and disease severity differed significantly between control subjects and patients with liver cirrhosis of all Child–Pugh classes (e.g., MELD score: 6.3 ± 0.7 in control subjects vs. 11.5 ± 4.9 in cirrhotic patients, *P* < 0.001). Detailed clinical characteristics of patients with liver cirrhosis and control subjects are given in Table 1.

**Table 1:** Clinical, laboratory and quantitative magnetic resonance imaging (MRI) parameters of control subjects and patients with liver cirrhosis of different Child–Pugh classes.

Variable	Controls (n = 22)	Child–Pugh A (n = 27)	Child–Pugh B (n = 32)	Child–Pugh C (n = 9)	*P* value
**Clinical parameters**					
Age (years)	44.7 ± 16.3^†^	48.4 ± 13.5^†^	60.6 ± 9.7*^‡^	57.9 ± 13.1	< 0.001
Body mass index (kg/m^2^)	25.6 ± 5.0	24.7 ± 2.9	23.6 ± 3.9	25.7 ± 5.4	0.271
Sex					0.102
Male	14 (64%)	18 (67%)	18 (56%)	5 (56%)	
Female	8 (36%)	9 (33%)	14 (44%)	4 (44%)	
**Underlying liver disease**					**0.005**
Autoimmune liver disease	0 (0%)	10 (37%)	5 (16%)	1 (11%)	
Alcoholic liver disease	0 (0%)	5 (18%)	17 (53%)	4 (44%)	
Viral hepatitis	0 (0%)	4 (15%)	4 (12%)	0 (0%)	
Non-alcoholic fatty liver disease	0 (0%)	0 (0%)	1 (3%)	2 (22%)	
Unknown	0 (0%)	7 (26%)	5 (16%)	2 (22%)	
Budd-Chiari syndrome	0 (0%)	1 (4%)	0 (0%)	0 (0%)	
**Laboratory parameters**					
Blood hematocrit level (%)	41.6 ± 3.9^†‖^	37.9 ± 0.7^†‖^	30.7 ± 0.5*^‡^	27.6 ± 0.8*^‡^	< 0.001
Bilirubin (mg/dl)	0.78 ± 0.51^†‖^	1.02 ± 0.49^‖^	1.89 ± 2.64*	2.97 ± 2.25*^‡^	< 0.001
ALT (U/l)	35.0 ± 11.2	49.1 ± 40.4	35.8 ± 26.9	31.6 ± 15.9	0.276
AST (U/l)	27.9 ± 15.4^‡†‖^	62.6 ± 44.7*	65.7 ± 41.3*	60.6 ± 9.7*	< 0.001
GGT (U/l)	33.5 ± 19.0^‡†‖^	198.2 ± 184.9*	178.5 ± 252.9*	148.7 ± 145.7*	< 0.001
AP (U/l)	50.5 ± 21.5^‡†‖^	161.6 ± 118.3*	161.0 ± 182.1*	166.0 ± 99.6*	< 0.001
Albumin (g/l)	49.2 ± 19.2	40.7 ± 5.9^†‖^	30.3 ± 9.7^‡^	26.8 ± 11.6^‡^	< 0.001
Platelets cells × 10^9^/l	282.7 ± 107.2^‡†‖^	174.9 ± 107.7*	151.4 ± 108.1*	113.0 ± 67.2*	< 0.001
International normalized ratio	1.03 ± 0.12^†‖^	1.12 ± 0.12^‖^	1.2 ± 0.2*	1.54 ± 0.64*^‡^	< 0.001
Creatinine (mg/dl)	0.86 ± 1.18^‖^	1.07 ± 0.95^‖^	1.13 ± 0.54	1.5 ± 0.6*^‡^	0.019
C-reactive protein level (mg/l)	1.4 ± 1.6^‡†‖^	7.3 ± 7.6*	12.6 ± 14.5*	15.3 ± 11.4*	< 0.001
MELD	6.3 ± 0.7^‡†‖^	9.3 ± 4.1*^†‖^	11.9 ± 4.2*^‡^	17.9 ± 6.1*^‡^	< 0.001
FIB-4	0.73 ± 0.47^‡†‖^	3.51 ± 3.68*^†^	6.15 ± 4.06*^‡^	6.53 ± 2.08*	< 0.001
APRI	0.22 ± 0.07^‡†‖^	1.18 ± 1.28*	1.36 ± 1.01*	1.56 ± 0.64*	< 0.001
**MRI parameters**					
Hepatic native T1 relaxation time (ms)	518.6 ± 47.9^‡†‖^	620.5 ± 78.9*^‖^	666.6 ± 73.4*^‖^	828.4 ± 91.2*^‡†^	< 0.001
Extracellular volume fraction (%)	25.9 ± 4.5^‡†‖^	33.3 ± 6.0*^†‖^	39.6 ± 4.9*^‡‖^	52.8 ± 1.2*^‡†^	< 0.001

Continuous variables are given as means ± standard deviations. Nominal data are absolute frequencies with percentages in parentheses. *P* values were obtained using ANOVA test followed by Turkey’s multiple comparison test.

*MELD* score model of end-stage liver disease, *ALT* alanine aminotransferase, *AST* aspartate aminotransferase, *AP* alkaline phosphatase, *GGT* gamma-glutamyltransferase, *APRI* aspartate aminotransferase to platelet ratio index, *FIB-4* fibrosis-4-score.

*P < 0.05 versus controls.

^‡^*P* < 0.05 versus Child–Pugh A.

^†^*P* < 0.05 versus Child–Pugh B.

^‖^*P* < 0.05 versus Child–Pugh C.

### MRI results

**Figure 1:**
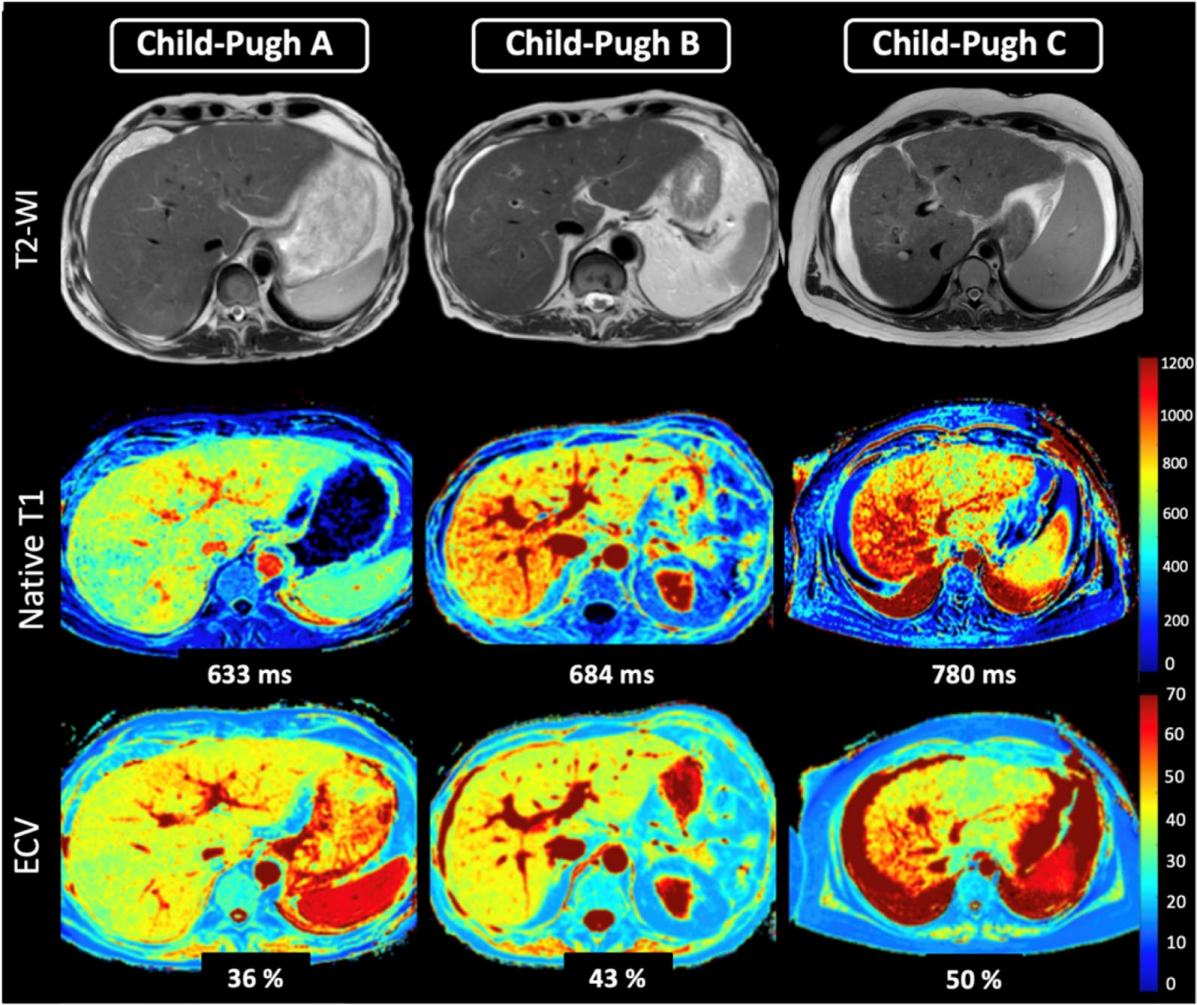
Representative images of T2-weighted images, hepatic native T1 and extracellular volume fraction (ECV) maps from a 54-years-old male patient with liver cirrhosis Child–Pugh class A, from a 61-year-old female patient with Child–Pugh class B, and a 41-year-old female patient with Child–Pugh class C. T1 relaxation times and ECV show increased values depending on Child–Pugh class. *T2-WI* T2-weighted image, *ECV* extracellular volume fraction.

**Figure 2:**
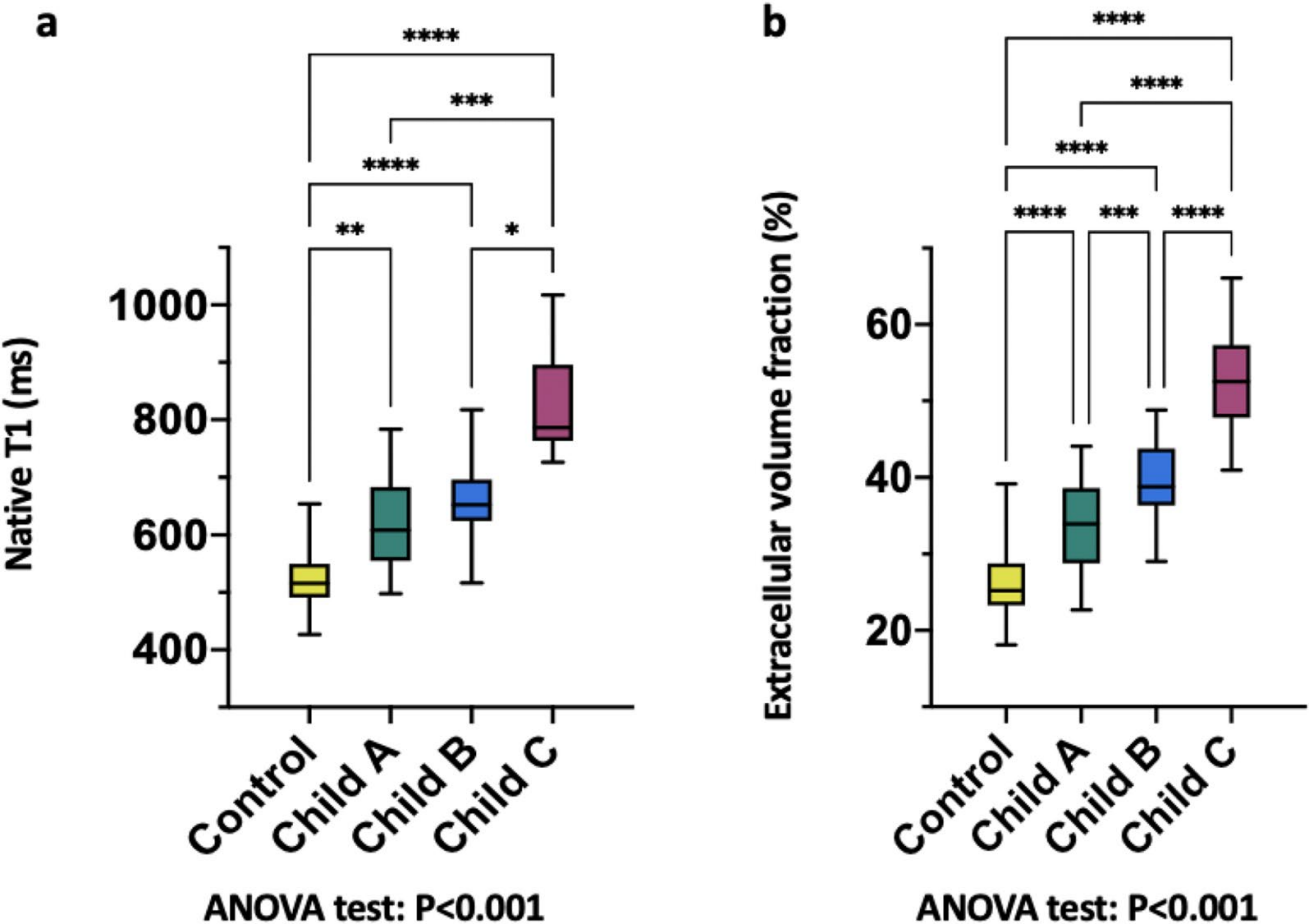
Column graphs with values distribution of hepatic native T1 (**a**) and MRI-derived extracellular volume fraction (**b**) in the control group and in the clinically subclassified cirrhosis groups (Child–Pugh classes A, B, and C). Mean of data is represented by horizontal line. *, **, ***, **** represents significance levels of pairwise comparisons with *P* values of ≤ 0.05, ≤ 0.01, ≤ 0.001, ≤ 0.0001respectively. *P* values were obtained using ANOVA test followed by Turkey’s multiple comparison test.

**Figure 3:**
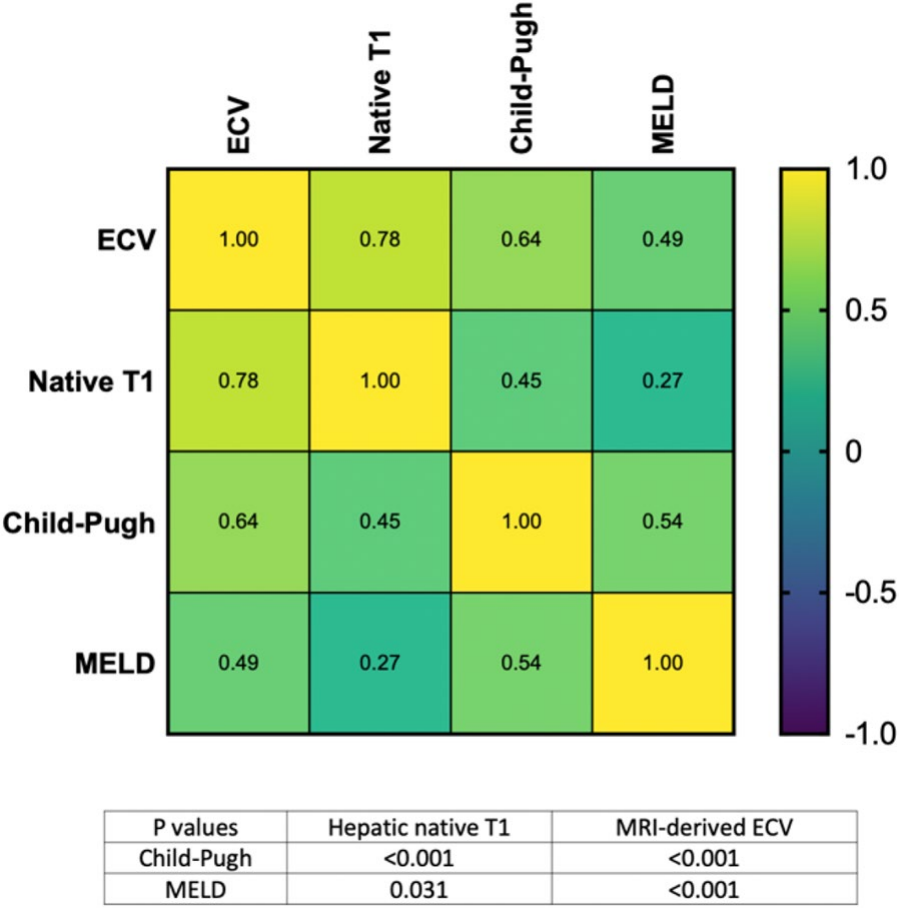
Heatmap shows correlations between hepatic native T1 and MRI-derived extracellular volume fraction (ECV) and clinical scores of liver disease severity. *ECV* extracellular volume fraction, *MELD* model for end-stage liver disease.

Hepatic T1 relaxation times were significantly higher in cirrhotic patients than in control subjects (518.6 ± 47.9 ms) and also increased depending on Child–Pugh class: 620.5 ± 78.9 ms (Child–Pugh A) vs. 666.6 ± 74.3 ms (Child–Pugh B) vs. 828.4 ± 91.2 ms (Child–Pugh C) (*P* < 0.001). Hepatic ECV values were also significantly higher in cirrhotic patients compared to control subjects (25.9 ± 4.5%) and increased depending on Child–Pugh class: 33.3 ± 6.0% (Child–Pugh A) vs. 39.6 ± 4.9% (Child–Pugh B) vs. 52.8 ± 1.2% (Child–Pugh C) (*P* < 0.001). There were also significant differences in hepatic ECV between patients with liver cirrhosis of different Child–Pugh classes: Child–Pugh A vs. B (33.3 ± 6.0% vs. 39.6 ± 4.9%, *P* < 0.001), A vs. C (33.3 ± 6.0% vs. 52.8 ± 1.2%, *P* < 0.001), and B vs. C (39.6 ± 4.9% vs. 52.8 ± 1.2%, *P* < 0.001) (see also Fig. 1). Hepatic MRI parameters of all included patients are given in Table 1, see also Fig. 2. According to correlation analysis, hepatic native T1 (r = 0.45, *P* < 0.001) and ECV (r = 0.64, *P* < 0.001) correlated with Child–Pugh score. A correlation matrix is given in Fig. 3.

### Diagnostic performance of MRI-derived mapping parameters

**Figure 4:**
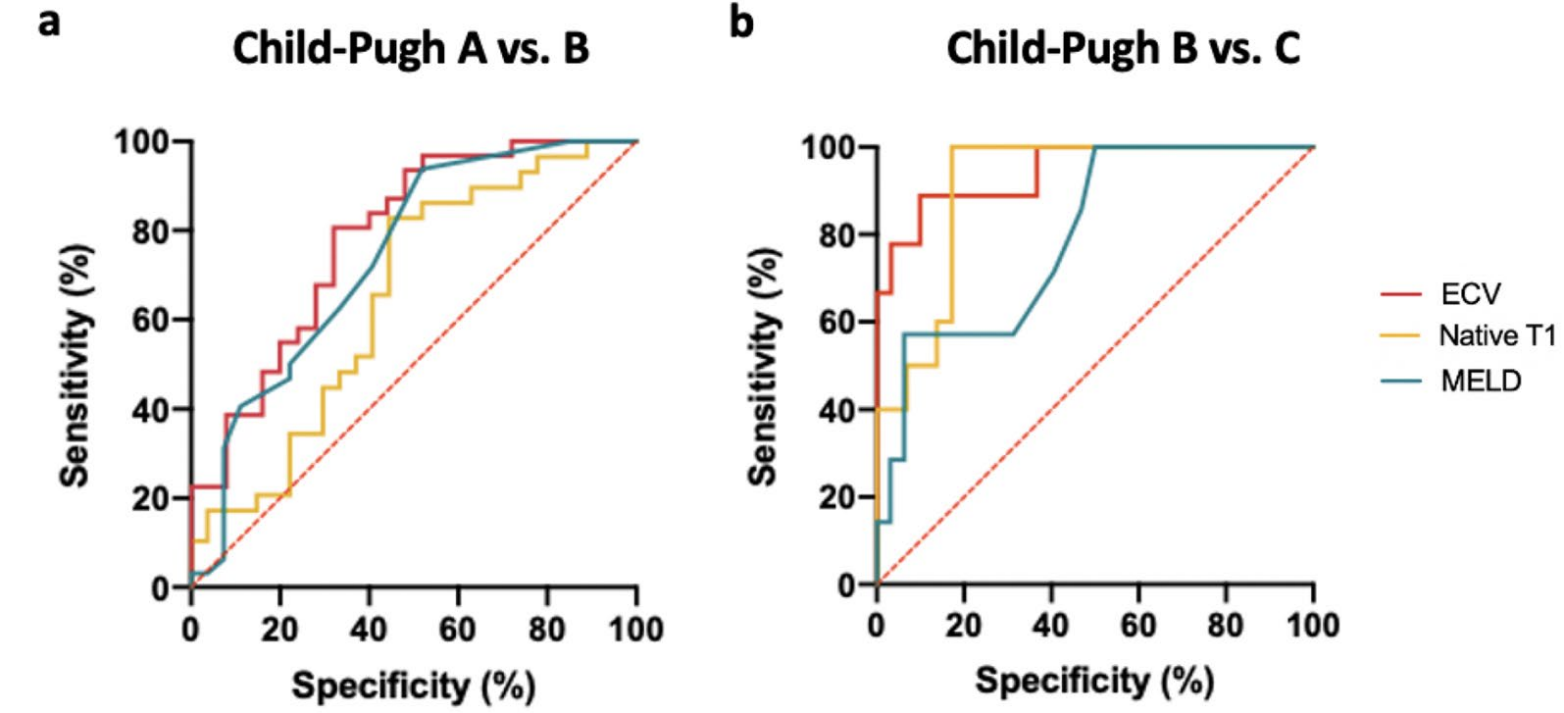
Graphs show receiver operating characteristic curves of hepatic native T1 and MRI-derived extracellular volume fraction (ECV) as well as clinical scores of liver disease severity for differentiation between different Child–Pugh A and B classes (**a**) and Child–Pugh B and C classes (**b**). (**a**) Curves are given for hepatic native T1 (area under the curve [AUC]: 0.651), hepatic ECV (AUC: 0.785), MELD (AUC: 0.740). (**b**) Curves are given for hepatic native T1 (AUC: 0.910), hepatic ECV (AUC: 0.944), MELD (AUC: 0.795). *ECV* extracellular volume fraction, *MELD* model of end-stage liver disease.

MRI-derived mapping parameters, as well as clinical scores of liver disease severity, were evaluated regarding their diagnostic performance to discriminate between different Child–Pugh classes. In general, the diagnostic performance of mapping parameters and MELD score were higher in discriminating between Child–Pugh classes B and C, than between Child–Pugh classes A and B (see also Tables 2, 3, and Fig. 4). Hepatic ECV revealed the highest diagnostic performance for differentiation between Child–Pugh classes A and B, as well as B and C, with an AUC of 0.785 (cutoff value: > 36.2%, sensitivity of 86.2%, specificity of 55.6%) and 0.944 (cutoff value: > 46.9%, sensitivity of 88.9%, specificity of 90%), respectively. The diagnostic performance of hepatic native T1 relaxation times was lower than that of ECV for differentiating between Child–Pugh scores A and B as well as between Child–Pugh score B and C with an AUC of 0.651 (cutoff: > 620.3 ms, sensitivity of 86.2%, specificity of 55.6%) and 0.910 (cutoff: > 722 ms, sensitivity of 100%, specificity of 82.8%) (*P* < 0.05, respectively). Furthermore, the diagnostic performance of native hepatic T1 relaxation times was higher than that of MELD score in differentiating between Child–Pugh classes B and C (0.910 vs. 0.795), but lower than that of MELD in differentiating between classes Child–Pugh A and B (0.651 vs. 0.740) (*P* < 0.05, respectively). Detailed parameters of diagnostic performance statistics are given in Tables 2 and 3.

**Table 2:** Diagnostic performance of hepatic native T1 and MRI-derived extracellular volume fraction as well as clinical scores of liver disease severity for the differentiation between patients with liver cirrhosis of Child–Pugh classes A and B.

	AUC	Cutoff value	Sensitivity (%)	Specificity (%)	PPV (%)	NPV (%)	Accuracy (%)
Native T1	0.651	> 620.3 ms	86.2 (69.4–94.5)	55.6 (37.3–72.4)	67.6 (51.5–80.4)	78.9 (56.7–91.5)	71.4 (58.5–81.6)
ECV	0.785	> 36.18%	80.6 (63.7–90.8)	68.0 (48.4–82.8)	75.8 (59.0–87.2)	73.9 (53.5–87.5)	75.0 (62.3–84.5)
MELD score	0.740	> 8.5	75.0 (57.9–86.7)	59.3 (40.7–75.5)	68.6 (55.1–78.3)	66.7 (46.7–82.0)	67.8 (55.1–78.3)
APRI score	0.618	> 0.786	68.8 (51.4–82.0)	51.9 (34.0–69.3)	62.9 (46.3–76.8)	58.3 (38.8–75.5)	61.0 (48.3–72.4)
FIB-4 score	0.760	> 3.242	84.4 (68.2–93.1)	63.0 (44.2–78.5)	73.0 (57.0–84.6)	77.3 (56.6–89.9)	74.6 (62.2–83.9)

*ECV* extracellular volume fraction, *MELD* model of end-stage liver disease, *APRI score* aspartate aminotransferase to platelet ratio index, *FIB-4 score* fibrosis 4 score, *AUC* area under the curve, *PPV* positive predictive value, *NPV* negative predictive value.

**Table 3:** Diagnostic performance of hepatic native T1 and MRI-derived extracellular volume fraction as well as clinical scores of liver disease severity for the differentiation between patients with liver cirrhosis of Child–Pugh classes B and C.

	AUC	Cutoff value	Sensitivity (%)	Specificity (%)	PPV (%)	NPV (%)	Accuracy (%)
Native T1	0.910	> 722 ms	100.0 (72.2–100.0)	82.8 (65.5–94.2)	66.7 (41.7–84.8)	100 (86.2–100.0)	87.2 (73.3–94.4)
ECV	0.944	> 46.85%	88.9 (56.5–98.0)	90.0 (74.4–96.5)	72.7 (43.4–90.3)	96.4 (82.3–99.4)	89.7 (76.4–95.9)
MELD score	0.795	> 10.5	100.0 (64.6–100.0)	50.0 (33.6–66.4)	30.4 (15.6–50.9)	100.0 (80.6–100.0)	59.0 (43.4–72.9)
APRI score	0.634	> 1.176	71.4 (35.9–91.8)	56.3 (39.3–71.8)	26.3 (11.8–48.8)	90.0 (69.9–97.2)	59.0 (43.4–72.9)
FIB-4 score	0.607	> 5.208	85.7 (48.7–97.4)	59.4 (42.3–74.5)	31.6 (15.4–54.0)	95.0 (76.4–99.1)	64.1 (48.4–77.3)

*ECV* extracellular volume fraction, *MELD* model of end-stage liver disease, *APRI score* aspartate aminotransferase to platelet ratio index, *FIB-4 score* fibrosis 4 score, *AUC* area under the curve, *PPV* positive predictive value, *NPV* negative predictive value.

## Discussion

The aim of this study was to evaluate the diagnostic utility of MRI-derived hepatic ECV for the assessment of cirrhosis severity as well discrimination of different Child–Pugh classes in patients with liver cirrhosis of various etiologies. The main findings of our study are: (1) hepatic native T1, as well as MRI-derived ECV, showed significant correlations with the Child–Pugh score and, (2) MRI-derived hepatic ECV revealed a high diagnostic performance for the discrimination of different Child–Pugh classes, which was higher than that of hepatic native T1 and MELD score.

The assessment of liver cirrhosis severity is currently based mainly on clinical and laboratory examinations with calculation of different scores, including the Child–Pugh score, as the most established one. In the past decade, different imaging modalities, including MRI, experienced a fast evolution and now represent an important pillar in terms of clinical management, risk stratification, prognosis estimation, and procedural planning in patients with CLD and cirrhosis. Elastography methods, including ultrasound- and MR-based elastography have become important diagnostic tools in patients with CLD, mainly for the assessment of fibrosis stage^21^. However, as the stage of liver fibrosis in cirrhotic patients is already final, the assessment of the fibrosis stage alone seems to be insufficient to draw conclusions about the liver function and disease severity. There are also studies demonstrating the ability of baseline liver stiffness measurements, as well as the dynamic of liver stiffness changes, for the prediction of hepatic decompensation^22–24^. In a cross-sectional setting, several MRI techniques have been tried out to assess the functional aspect of liver cirrhosis/disease, e.g., using DWI extended to intravoxel incoherent motion, contrast-enhanced T1 techniques using different techniques and contrast media (e.g., hepatocyte-specific vs. extracellular), and even T1 rho mapping. However, these techniques suffer from lack of standardization (e.g., DWI and contrast-enhanced MRI) and availability across institutions (e.g., T1 rho mapping). Quantitative MRI mapping using T1 mapping techniques with calculation of ECV may potentially overcome these limitations and allow for assessment of liver function and disease severity. A representative hepatic T1 map can be acquired during a single breath-hold and T1 values can be fast and directly obtained from the parametric map. Therefore, the technique can be implemented cost-effectively into clinical routine. It is known that fibrosis is associated with prolongation of T1 relaxation times (which can be also caused due to intra- and extracellular edema in inflammatory settings). Also, fibrosis is associated with an expansion of extracellular space and, as a consequence, with an increased accumulation of extracellular contrast in the extracellular space, which is reflected in increased ECV values^13–16^.

In our study we extended the applicability of mapping techniques to the assessment of liver cirrhosis severity. We found a significant correlation between hepatic native T1 and Child–Pugh score (r = 0.45). This is consistent with some previous studies, showing that cirrhotic changes lead to prolongation of T1 relaxation times compared to healthy subjects and increase with the increasing stage of liver cirrhosis from patients with Child–Pugh A up to C^25,26^. However, there are other studies, showing no significant differences in native T1 relaxation times between healthy volunteers and cirrhotic patients^11,27^. These conflicting results have been discussed controversially. On the one hand, prolongation of hepatic native T1 relaxation times could be explained by the tissue remodeling, on the other hand, shortening of the T1 relaxation times in patients with liver cirrhosis may be explained by the presence of paramagnetic molecules (e.g. iron) as well as the presence of macromolecules with increased amounts of bound water^11,25,26,28–32^. Liver function might also be correlated with post-contrast hepatic T1 relaxation times in patients with liver cirrhosis. However, post-contrast T1 relaxation times of the liver are highly variable as they depend on time and flow rate of contrast agent application, as well as the applied contrast agent (e.g. hepatocyte-specific vs. extracellular). Furthermore, post-contrast values may vary depending on contrast agent dose, renal clearance rate, time, as well as hematocrit level. These factors would limit the general applicability and a comparability of study results.

Unlike native and post-contrast T1 relaxation times, ECV seems to be a physiologically normalized and a more robust parameter as it does not depend on magnetic field and acquisition parameters. Expansion of the extracellular matrix caused by chronic liver injury leads to enlargement of the extracellular space and, consequently to increased ECV values^15,16,33^. According to histopathological studies, collagen proportionate area increases proportionately across all stages of cirrhosis, which can be explained by the fact that thicker cirrhotic septa contain more collagen^34^. Increased hepatic ECV values in liver cirrhosis, may reflect increased extracellular matrix protein synthesis and deposition, which is higher in advanced stages. For the same reason, ECV is well-known parameter in cardiac MRI and can be employed for non-invasive assessment of myocardial fibrosis^35–37^. There are also studies in animal and humans, demonstrating that ECV correlates better with portal pressure measurements than native T1^16,38^. In our study, we demonstrated significant differences in ECV values between all Child–Pugh classes, which was also different to that in the healthy subjects. ECV correlated stronger with Child–Pugh score than hepatic native T1 (r = 0.64 vs. 0.45). Our study results also support previous data in terms of diagnostic utility of ECV to diagnose liver cirrhosis^12–14,39,40^. However, none of the previous study focused exclusively on cirrhotic patients, nor on the ability of MRI-derived ECV to differentiate between different cirrhosis classes.

Finally, we demonstrated a high diagnostic performance of mapping parameters to discriminate between different cirrhosis classes, which was also higher than that of the MELD score. This might be explained by the fact that for the calculation of clinical scores of liver disease severity different laboratory and clinical markers are used. On the one hand, it may decrease the specificity of these markers, as changes outside the liver and also comorbidities, which are not primary related to liver disease, contribute to the final score. On the other hand, ECV seems to be especially more liver-specific as all variables for ECV calculation are obtained from liver parenchyma directly^41^ and then normalized for hematocrit. Moreover, approaches for automated calculation of ECV even without hematocrit sampling already exist and can be further developed with the use of machine learning^41,42^. However, because clinical information and laboratory markers are crucial for the assessment of liver function and disease severity, the intention of this study was not to discourage the use of clinical scores and laboratory markers but instead to demonstrate the potential diagnostic value of a quantitative imaging approach.

Despite the advantages of MRI-derived ECV as a potential non-invasive biomarker of liver cirrhosis severity, our study has several limitations. First, the small sample size with a limited number of controls and patients with Child–Pugh class C limit the generalizability of the the study results. Second, we included patients with CLD and cirrhosis of different etiologies. This may have an influence on hepatic T1 values, as the pattern of liver fibrosis and cirrhosis depends on underlying etiology of CLD. Another limitation is the absence of liver biopsy as the reference standard at the time of MRI examination. Larger prospective studies focusing on the etiology of liver disease in correlation with histopathological findings are needed to further investigate the diagnostic utility of MRI-derived ECV.

In conclusion, this is the first study investigating the diagnostic utility of MRI-derived ECV for the assessment of cirrhosis severity. MRI-derived ECV can provide valuable diagnostic information beyond standard morphological imaging for liver fibrosis assessment and might represents a new non-invasive imaging-based biomarker for the assessment and follow-up of liver cirrhosis severity. Our study results might also motivate future studies to evaluate whether quantitative liver MRI can be used in combination with clinical scoring to improve severity assessment and outcome prediction in patients with liver cirrhosis.

## Data Availability

The datasets generated during and/or analyzed during the current study are available from the corresponding author on reasonable request.
